# Analysis of structure and function of the giant protein Pf332 in *Plasmodium falciparum*

**DOI:** 10.1111/j.1365-2958.2008.06508.x

**Published:** 2008-11-06

**Authors:** Anthony N Hodder, Alexander G Maier, Melanie Rug, Monica Brown, Mirja Hommel, Ivan Pantic, Marina Puig-de-Morales-Marinkovic, Brian Smith, Tony Triglia, James Beeson, Alan F Cowman

**Affiliations:** 1 The Walter and Eliza Hall Institute of Medical ResearchMelbourne, Australia; 2 Department of Environmental Health, Harvard School of Public HealthBoston, MA 02115, USA

## Abstract

Virulence of *Plasmodium falciparum*, the most lethal parasitic disease in humans, results in part from adhesiveness and increased rigidity of infected erythrocytes. Pf332 is trafficked to the parasite-infected erythrocyte via Maurer's clefts, structures for protein sorting and export in the host erythrocyte. This protein has a domain similar to the Duffy-binding-like (DBL) domain, which functions by binding to receptors for adherence and invasion. To address structure of the Pf332 DBL domain, we expressed this region, and validated its fold on the basis of the disulphide bond pattern, which conformed to the generic pattern for DBL domains. The modelled structure for Pf332 DBL had differences compared with the erythrocyte-binding region of the αDBL domain of *Plasmodium knowlesi* Duffy-binding protein (Pkα-DBL). We addressed the function of Pf332 by constructing parasites that either lack expression of the protein or express an altered form. We found no evidence that Pf332 is involved in cytoadhesion or merozoite invasion. Truncation of Pf332 had a significant effect on deformability of the *P. falciparum*-infected erythrocyte, while loss of the full protein deletion did not. Our data suggest that Pf332 may contribute to the overall deformability of the *P. falciparum*-infected erythrocyte by anchoring and scaffolding.

## Introduction

Malaria is a major disease in humans with over 500 million cases of infection and 1–3 million deaths every year (Snow *et al*., 2005). *Plasmodium falciparum* is the protozoan parasite responsible for the severe form of this disease causing nearly all malaria deaths and most of the morbidity. The blood stage of the parasite infects circulating erythrocytes. Once inside it initiates a remarkable remodelling process that converts a terminally differentiated cell lacking fundamental structures and functions into a host in which the parasite can grow and survive (see for review Marti *et al*., 2005). The properties of the *P. falciparum*-infected erythrocytes are dramatically altered becoming more rigid and imparting the ability of the host cell to adhere to endothelial and other cell types. The altered properties of the parasite-infected red cells play an important role in pathogenesis of malaria (see for review Miller *et al*., 2002).

The properties of *P. falciparum*-infected erythrocytes are mediated by parasite proteins exported into the host cell and deposited beneath and onto the host cell membrane. These proteins are trafficked in a multistep process requiring transfer across several membranes, including the parasite and parasitophorous vacuole membrane, and the erythrocyte membrane after transport through the host cell cytoplasm (Marti *et al*., 2005). Trafficking of proteins beyond the parasitophorous membrane requires these molecules to associate with membranous structures in the *P. falciparum*-infected erythrocytes called Maurer's clefts. These structures bud from the parasitophorous vacuole membrane and transport proteins to the periphery of the erythrocyte allowing sorting and transfer to the underside of the erythrocyte membrane and, for some proteins, insertion into the host cell membrane (Waterkeyn *et al*., 2000; Wickham *et al*., 2001; Lopez-Estrano *et al*., 2003). The surface of the parasite-infected erythrocyte becomes covered with elevations called knobs, which consist primarily of the knob-associated histidine-rich protein (KAHRP) (Kilejian, 1979; Culvenor *et al*., 1987). Adherence of *P. falciparum*-infected erythrocytes is mediated by *P. falciparum* erythrocyte membrane protein 1 (PfEMP1) (Leech *et al*., 1984), a large and antigenically diverse protein family that is expressed after invasion of the erythrocyte. It is trafficked to the host cell surface via Maurer's clefts before being concentrated on the knob structures (Baruch *et al*., 1995; Smith *et al*., 1995; Su *et al*., 1995).

Pf332 is a large protein of approximately 700 kDa that localizes to Maurer's clefts and has been reported to be trafficked to the surface of the *P. falciparum*-infected erythrocyte (Mattei and Scherf, 1992; Mattei *et al*., 1992). This protein has a Duffy-binding-like (DBL) domain at the N-terminus followed by a putative transmembrane region and a very large C-terminus that is highly charged and repetitive with respect to the amino acid sequence (Moll *et al*., 2007). It has been suggested that Pf332 plays a role in merozoite invasion as the DBL domain has been expressed in Chinese hamster ovary (CHO) cells and *Escherichia coli* and was shown to bind erythrocytes (Moll *et al*., 2007) and antibodies to the DBL domain inhibited invasion in a number of *P. falciparum* strains (Ahlborg *et al*., 1996). Antibodies to the C-terminal region have also been shown to inhibit *in vitro* growth of *P. falciparum* although in this case they appeared to interfere with late-stage parasite development and the effect was likely due to cross-reaction with other proteins (Ahlborg *et al*., 1996).

The DBL domain is *Plasmodium* spp. specific shared by a number of protein families that include the DARC (Duffy antigen receptor for chemokines)-binding protein from *Plasmodium knowlesi* (Pkα-DBL). The erythrocyte-binding-like proteins (ebl) of *P. falciparum* also contain DBL domains (Adams *et al*., 2001) and this family consists of EBA-175, EBA-181 (also known as JESEBL) and EBA-140 (also known as BAEBL), which are proteins that play a key role in invasion of the merozoite form of the parasite into human erythrocytes by binding to specific host receptors (see for review Cowman and Crabb, 2006). The three-dimensional structures of the DBL domains from EBA-175 and Pkα-DBL have been solved (Tolia *et al*., 2005; Singh *et al*., 2006).

Here we have expressed and refolded the DBL domain of Pf332 and determined the disulphide topology using a combination of reversed-phase (RP)-HPLC peptide mapping under reducing and non-reducing conditions and subsequent Edman degradation and mass spectroscopic analysis of the identified disulphide-linked peptides. These data have been used to validate a modelled structure of Pf332 DBL domain, which significantly differs in the region corresponding to the site identified in Pkα-DBL that binds to DARC on erythrocytes (Choe *et al*., 2005). We also describe targeted mutagenesis and disruption of the *Pf332* gene to test its role in cytoadherence and merozoite invasion and deformability.

## Results

### Expression and oxidative in vitro refolding of the recombinant Pf332 DBL domain

**Fig. 1:**
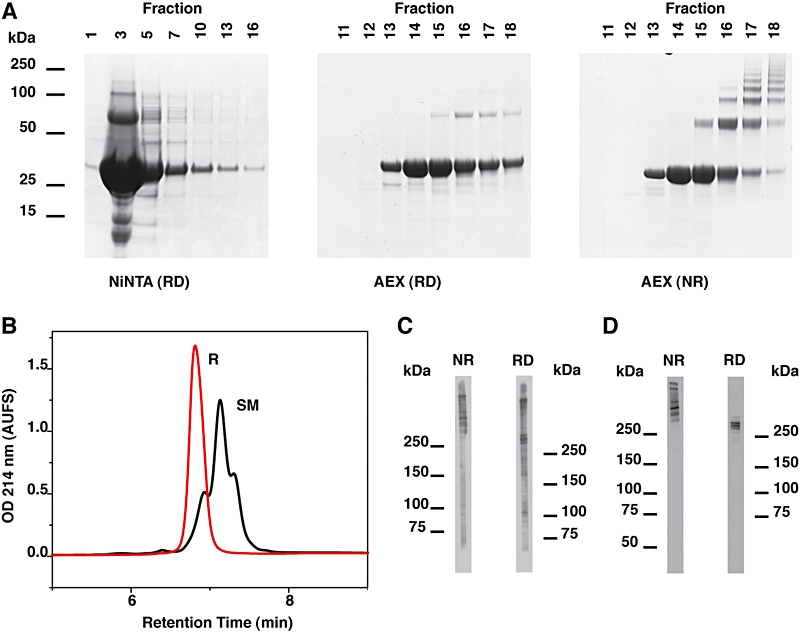
Production and characterization of the recombinant Pf332 DBL domain. A. SDS-PAGE analysis of denatured Pf332 DBL domain solubilized from inclusion bodies then purified using NiNTA agarose. After the oxidative, *in vitro* refolding process, correctly refolded Pf332 DBL domain (AEX fractions #13 and #14) was separated from soluble multimers by anion-exchange chromatography (AEX). All samples shown in (A) were electrophoresed in sample buffer with (RD) or without (NR) reducing agent as indicated. B. RP-HPLC analysis of the denatured starting material (SM) and the refolded Pf332 DBL domain (R). A decreased retention time is consistent with protein refolding. AUFS, Absorbance units full scale. C and D. Immunoblots for saponin-lysed *P. falciparum* 3D7 strain parasites were probed with (C) polyclonal mouse serum and (D) monoclonal antibody 10H2 each raised to the refolded Pf332 DBL domain. Parasite samples were electrophoresed with (RD) or without (NR) reducing agent in the sample buffer, then transferred onto PVDF membrane prior to commencing immunoblots.

Under the cell culture conditions utilized, the cysteine-rich DBL domain of Pf332 was deposited in *E. coli* exclusively as inclusion bodies. A denaturing buffer, containing 6 M guanidine, was used to solubilize and extract the Pf332 DBL domain. The domain fragment was purified from whole-cell lysate by passage over NiNTA agarose resin, giving approximately 80–90% purity of the material in a single chromatographic step (Fig. 1A). The denatured domain fragment was then oxidatively refolded *in vitro* prior to purification using strong anion-exchange chromatography. Fractions collected from the ion exchange are shown (Fig. 1A). Only those fractions that contained the DBL domain monomer were pooled (e.g. fractions 13 and 14) as later eluting fractions contained covalent multimers, which occur as a by-product of the oxidative refolding process. Noteworthy is the differential migration of the Pf332 monomer when electrophoresed in the presence of reducing and non-reducing sample buffers. This observation is consistent with the monomer having a disulphide bond architecture which influences the binding of sodium dodecylsulphate (SDS) to Pf332 DBL domain, resulting in a faster rate of migration than that observed for the reduced material. RP-HPLC was used to demonstrate that a decrease in the DBL domain's hydrophobicity had occurred as a result of the *in vitro* refolding process. The refolded material eluted significantly earlier than the denatured starting material consistent with internalization of hydrophobic residues upon refolding (Fig. 1B). The monomeric form of the DBL domain for Pf332 was found to be quite stable for extended periods at 4°C hence indicating no reactive surface accessible Cys residues were present in the final product.

The refolded antigen was used to immunize rabbits and mice to produce polyclonal and monoclonal antibodies and their specificity was determined on saponin-lysed parasitized erythrocytes. Both rabbit and mouse polyclonal antibodies produced similar profiles on immunoblots and reacted with several very high-molecular-weight protein bands (> 250 kDa) when electrophoresed in the presence of reducing and non-reducing sample buffers on 3–8% Tris-acetate gels (Fig. 1C). Several monoclonal antibodies raised to the recombinant Pf332 DBL domain, including 10H2, also gave similar staining patterns on immunoblots as seen for the polyclonal sera, and it appears that the Pf332 parent molecule undergoes significant proteolytic breakdown in schizont-stage parasites (Fig. 1C and D). However, the monoclonal antibodies failed to significantly react against the two largest protein bands when samples were electrophoresed in reducing sample buffer. This observation is probably due to these monoclonal antibodies targeting a reduction-sensitive epitope that may reform in the lower-molecular-weight (∼250 kDa) species during electrophoresis.

### Determination of the disulphide-bond pattern within the recombinant DBL domain of Pf332

**Fig. 2:**
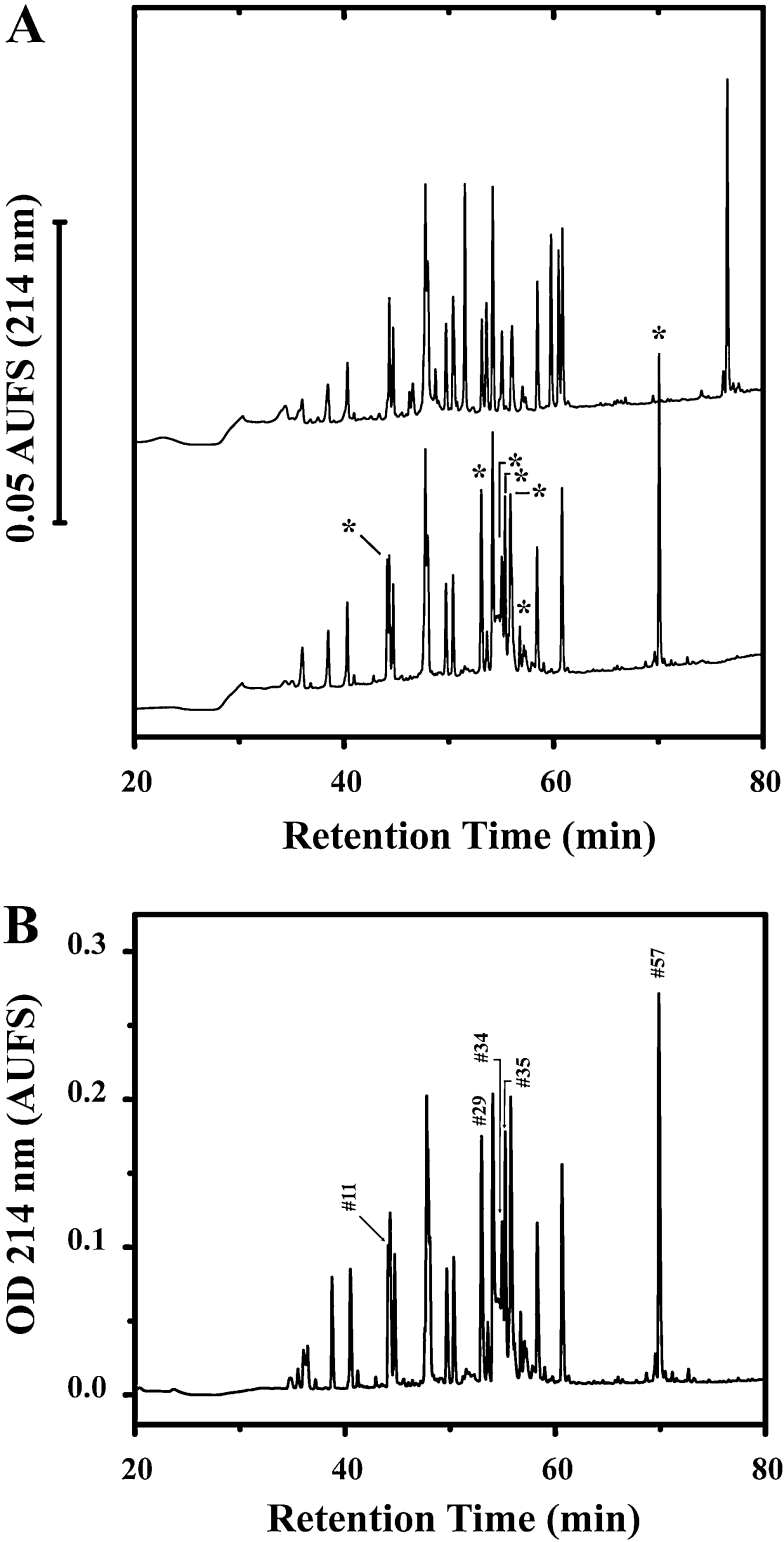
Identification and purification of disulphide-bonded peptides from a tryptic digest of Pf332 DBL domain. A. Comparison of the analytical RP-HPLC elution profiles for DTT-reduced and non-reduced tryptic digest of refolded Pf332 DBL domain. Reduced (upper trace) and non-reduced (lower trace) digests (20 μg) were fractionated on a Vydac C18 (4.6 mm inner diameter × 250 mm) column under identical chromatographic conditions. The column was developed at a flow rate of 1 ml min^−1^ with a linear 120 min gradient from 0 to 70% buffer B, where buffer A was 0.05% (v/v) trifluoroacetic acid in Milli Q water, and buffer B was 0.05% (v/v) trifluoroacetic acid in acetonitrile. Asterisk-labelled peaks within the non-reduced chromatogram represent those altered by DTT reduction. B. Preparative scale RP-HPLC purification of peptides from the tryptic digest of Pf332 DBL domain. A total of 125 μg of digest was fractionated by elution from a Vydac C18 column (4.6 mm inner diameter × 250 mm) column using the elution conditions described in (A). Peptide fractions found to contain disulphide-bonded peptides by Edman degradation and mass spectrometry are indicated by peak numbers and correspond to those given in Table 1 (see *Experimental procedures* for further details).

In order to further characterize the conformation of the Pf332 DBL domain the refolded protein was digested extensively in trypsin and disulphide-linked peptides were identified by comparison of analytical RP-HPLC profiles for DTT-reduced and non-reduced digests in a manner similar to that described previously (Hodder *et al*., 1996) (Fig. 2). Those peaks containing disulphide-linked peptides were identified by their altered chromatographic behaviour after reduction with DTT and are labelled with an asterisk (Fig. 2A). The procedure was then scaled up using the same column and elution conditions and the contents of the peaks of interest identified using a combination of Edman degradation and mass spectrometric analysis (Fig. 2B and Table 1). The disulphide linkages between 8 of the 12 Cys residues in the DBL domain were resolved without further sample processing; however, Cys-69, Cys-73, Cys-140 and Cys-144 were all found to be in a trypsin resistant tri-peptide bundle: the localization of Cys-69 and Cys-73 within the same peptide revealed that each of these residues was linked to an alternative Cys residue (either Cys-140 or Cys-144) within one of the other peptides. Interestingly, these four Cys residues are from subregion II in the DBL domain where only a single disulphide linkage has previously been determined (Singh *et al*., 2006). The presence of acidic residues between Cys-69 and Cys-73 enabled use of AspN protease to promote restricted cleavage specifically at Glu residues. Mass spectrometry was subsequently used to analyse the subdigest of peak fraction 34 and the remaining two disulphide-bond linkages were resolved by the observation of molecular ions that corresponded to the theoretical ion mass for the expected cleavage products of the known tri-peptide sequence. The disulphide-bond linkages determined in the Pf332 DBL domain are listed Table 1.

**Table 1:** Assignment of cysteine connectivities within the Pf322 DBL domain using N-terminal sequencing and ion-trap spectrometric data.

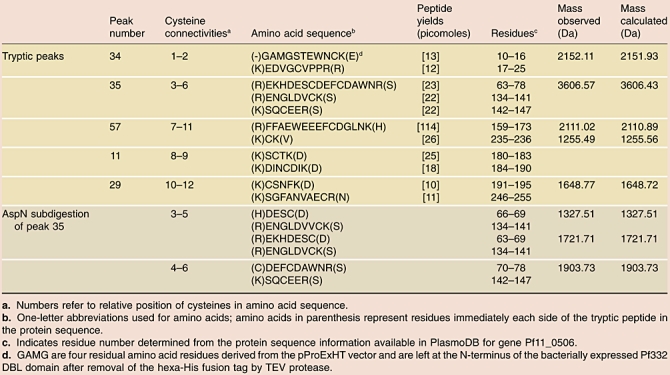

### A comparison of the disulphide-linkage pattern in the DBL domains for EBA-175, Pkα-DBL and Pf332

**Fig. 3:**
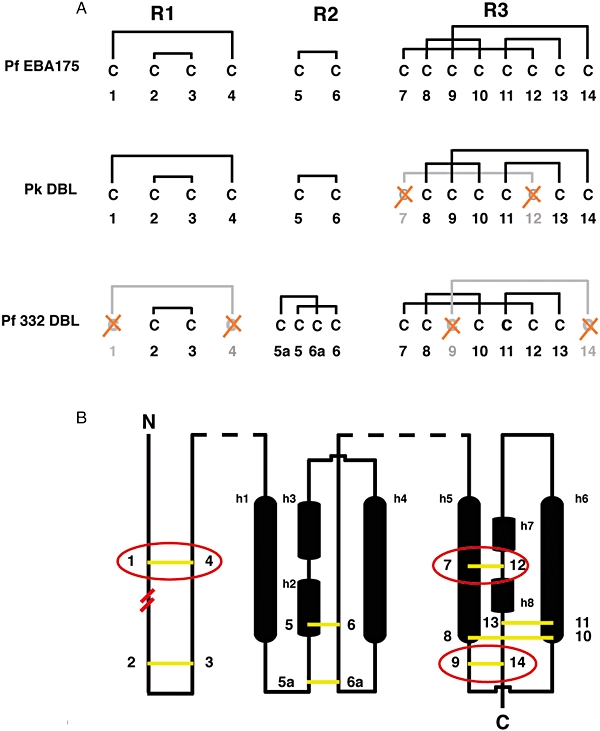
Analysis of the disulphide bond patterns within known DBL domains. A. Disulphide-bond linkage patterns for Pf EBA-175 F2, Pk DBP and Pf332 DBL domains. Cysteine residues are numbered relative to their occurrence in the protein sequence and are clustered by their location in either subregion 1, 2 or 3 (R1, R2, R3) in the DBL domain. Crossed (red) Cys residues have been lost from the sequence and result in the subsequent loss of the corresponding disulphide bond from the protein structure, relative to the EBA-175 F2 DBL domain. B. A two-dimensional schematic representation of the Pf EBA-175 F2 DBL domain showing the relative position of disulphide bonds (yellow) in Pf332 and Pk DBP DBL domains. Those disulphides lost from either structure are circled in red. The start of the Pf332 DBL sequence relative to the two other DBL domains is indicated by parallel red lines between the Cys-1 and Cys-2 residues. Structurally relevant helices are represented as black cylinders and are labelled h1–h8. Dashed lines (black) represent regions where sequence has not been considered. For the Pf332 DBL domain C2 = Cys-15, C3 = Cys-21, C5a = Cys-69, C5 = Cys-73, C6a = Cys-140, C6 = Cys-144, C7 = Cys-168, C8 = 181, C10 = 187, C11 = Cys-191, C12 = Cys-235 and C13 = Cys-254.

**Fig. 4:**
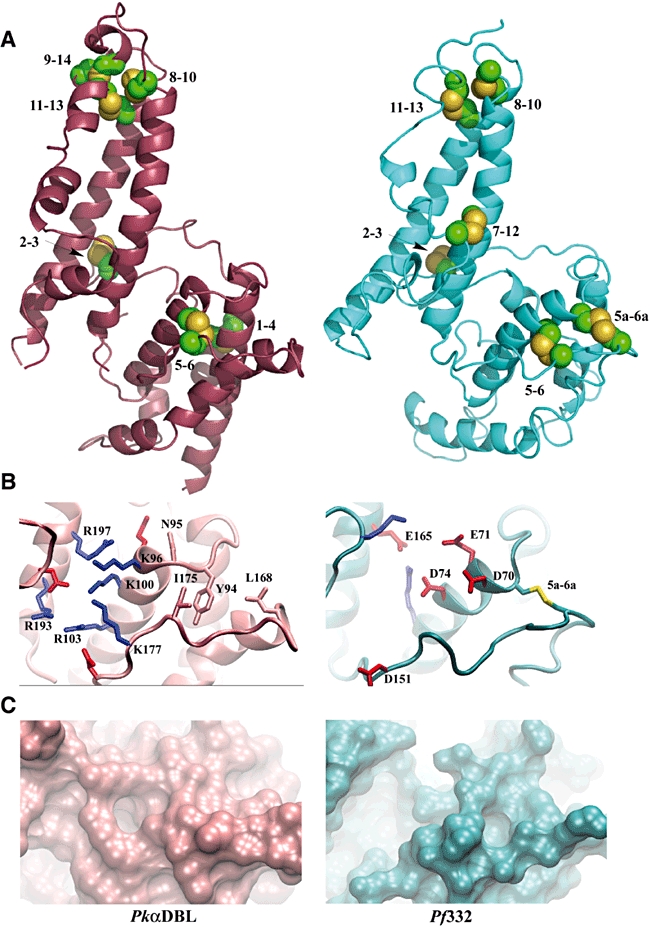
Analysis of the structure for the Pf332 DBL domain. A. Schematic representations of the structures for the DBL domains of Pkα-DBL (magenta) and that modelled for Pf332 (blue). The disulphide-bond pattern, determined via tryptic digestion of the refolded protein and from modelling the structure of Pf332 DBL domain, is compared with that found in the structure of Pkα-DBL (PDB 2C6J). Cysteine residues are numbered and displayed in yellow. B. A comparison of the DARC binding site from Pkα-DBL and the equivalent region in Pf332. Basic residues are shown in blue and acidic residues are shown in red. The four non-polar residues considered important for binding a sulphated tyrosine on the DARC receptor (Y94, N95, L168 and I175) are shown in the colour of the Pkα-DBL backbone. The Cys-5a–Cys-6a disulphide bond is shown in yellow. C. The surface topographies resulting from the differences in amino acid composition in the DARC binding site for Pkα-DBL domain and the equivalent region in the Pf332 DBL domain.

A molecular model of Pf332 DBL domain was produced using the EBA-175 structure as a template (Tolia *et al*., 2005). A similarity comparison of the Pf322 DBL and EBA-175 F2 domains revealed significant homology (Fig. S1). This structure enabled the comparison of the overall pattern and location of the disulphide bonds within the DBL domain of Pf332 with that of another well-characterized single DBL domain from *Plasmodium* involved in binding erythrocytes (Figs 3 and 4) (Tolia *et al*., 2005; Singh *et al*., 2006). The Pkα-DBL domain contains 12 Cys residues compared with 14 Cys residues in the EBA-175 F2 DBL domain. A structural comparison for these two proteins reveals that the disulphide-bond pattern is identical in both proteins, except that the Cys-7–Cys-12 linkage in subregion 3 is missing in Pkα-DBL. The disulphide-bond pattern for Pf332 was found to be similar but not identical to that found for the EBA-175 F2 DBL domain, with three significant differences observed. First, as for the Pkα-DBL domain, a disulphide bond was found to be missing from subregion 3; however, the Cys-9–Cys-14 linkage was lost in the Pf332 DBL domain instead of the Cys-7–Cys-12 disulphide bond. Second, the Cys-1–Cys-4 disulphide bond found in subregion 1 of both EBA-175 F2 and Pkα-DBL domains was not present in the Pf332 DBL domain (Fig. 3). The first and second cysteine residues (i.e. Cys-15 and Cys-21) in the mature sequence of Pf332 correspond to the Cys-2 and Cys-3 residues in EBA-175 F2 and Pkα-DBL domains (Figs 3 and 4). This conclusion is supported not only by their location in the modelled structure of the Pf332 DBL domain (Fig. 4), but also by their location relative to conserved residues in the protein sequence. For example, Trp-13 located two amino acid residues on the N-terminal side of Cys-15 and the Pro–Pro–Arg–Arg motif located on the C-terminal side of Cys-21. Third, an additional, previously unreported, disulphide linkage was determined between Cys-5a (Cys-69) and Cys-6a (Cys-140) in subregion 2. The existence of the disulphide bond between Cys-69 and Cys-140 was confirmed experimentally in the *in vitro* refolded recombinant DBL domain (Table 1) and was also predicted to occur between these two cysteines residues in the modelled structure. In this structure Cys-69 and Cys-140 were found to interact within a van der Waals radius of each other with the correct spatial orientation for disulphide bond formation (Fig. 4). The Cys-5a–Cys-6a linkage is located on the same face of the protein as the Cys-5–Cys-6 linkage.

### The residues in Pkα-DBL important for participating in binding to the DARC on erythrocytes are absent from the predicted interaction site in the Pf332 DBL domain

It is envisaged that two domains sharing a similar fold and the same function would interact with erythrocytes via a similar binding mechanism. In the Pkα-DBL domain (pink backbone), Tyr-94, Leu-168 and Ile-175 form a hydrophobic pocket to promote interaction with the DARC on erythrocytes (Choe *et al*., 2005). However, a hydrophobic pocket does not occur between juxtapositioned residues within the equivalent region of the Pf332 DBL domain (Fig. 4B). Interestingly, the additional disulphide bond between Cys-5a and Cys-6a was found to occur in the same area suggesting a restricted or stabilized conformation was required in this region of the DBL domains. Additionally, the essential polar residues involved in DARC recognition in Pkα-DBL (Asn-95, Lys-96 and Arg-103 –*Pk*α-DBL numbering) were also absent in Pf332.

The cluster of basic residues (Lys-100 and Lys-177 on subdomain II, and Arg-193 and Arg-197 on subdomain III – Pkα-DBL numbering) which also form the remaining part of the DARC binding site in Pkα-DBL domain, which may bind the sulphonated Tyr-41 of DARC (Choe *et al*., 2005), are not observed in the equivalent region of the Pf332 DBL structure (Fig. 4B). Instead an increased density of acidic residues (i.e. residues Glu-70, Glu-71, Asp-74, Glu-165 and Glu-151) was evident, which would be expected to present a very different surface polarity in this region compared with that found in Pkα-DBL (Singh *et al*., 2006). The surface topographies generated for the DARC binding site and the equivalent region in Pf332 varied significantly and resulted from quite different amino acid compositions in this region of the molecules (Fig. 4C). Particularly noteworthy was the formation of a ridge by the Cys-5a and Cys-6a disulphide bond through the area equivalent to the hydrophobic pocket in Pkα-DBL and the large cavity observed in Pf332 instead of the cluster of basic residues in Pkα-DBL.

### Disruption of the Pf332 gene in *P. falciparum*-infected erythrocytes

**Fig. 5:**
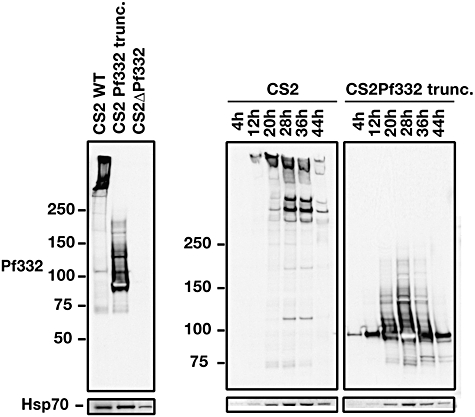
Western blot of saponin pellets of trophozoite-infected erythrocytes with CS2, CS2Pf332trunc and CS2ΔPf332 using monoclonal antibody 10H2 that is specific for the Pf332 DBL domain. Time-course across the asexual blood stage with equal number of parasites added to each lane showing maximal expression of Pf332 full length and the Pf332 truncation in trophozoite stages. Parasite pellets from synchronized CS2 parasites taken at 8 h intervals were separated by SDS-PAGE, transferred to nitrocellulose and probed with the mouse anti-Pf332 10H2 monoclonal antibody. Anti-PfHSP70 antibodies shown at the bottom of each panel was used to confirm correct development of the parasites across the asexual blood stage.

To analyse the function of Pf332 in *P. falciparum*, we disrupted the corresponding gene (PF11_0506) by double-cross-over homologous recombination (Fig. S2). In addition, we generated a second parasite line expressing a truncated form of the Pf332 (Fig. S2). Expression of the truncated protein in CS2Pf332trunc and absence of Pf332 in CS2ΔPf332 was confirmed using immunoblots with Pf332 monoclonal antibody 10H2 (Fig. 5). High-molecular-weight bands of approximately 700 kDa and 400 kDa were observed for cloned parental line CS2, whereas CS2Pf332trunc showed a major band of approximately 90 kDa, which corresponds to the predicted theoretical size of 87.3 kDa. There was no reactivity of the Pf332 antibody with CS2ΔPf332 confirming the gene disruption and demonstrating the specificity of this antibody.

The temporal expression pattern of Pf332 was determined using specific antibodies to show that both the full-length protein in CS2 and the truncated form in CS2Pf332trunc were expressed during the asexual blood stage in trophozoite stages. Immunoblot analysis with anti-Pf332 antibodies demonstrated that this protein was expressed primarily between 28 and 32 h post invasion, which corresponds to late trophozoite stages (Fig. 5D). This is consistent with the transcription pattern of Pf332 as demonstrated using microarray analysis (Bozdech *et al*., 2003; Le Roch *et al*., 2003) and previous analysis of Pf332 expression (Moll *et al*., 2007).

**Fig. 6:**
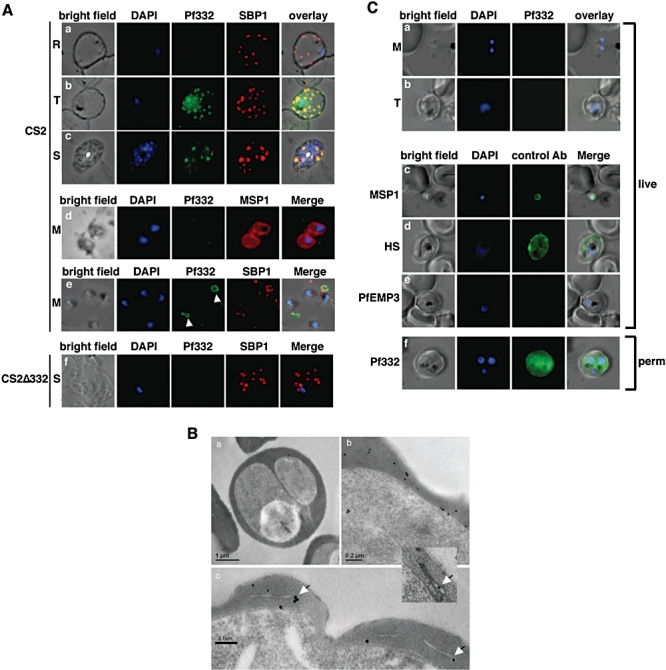
Detection of Pf332 in *P. falciparum* parasites throughout the blood-stage life cycle. A. Localization of Pf332 in ring stage-infected erythrocytes (R) (a), trophozoite-infected erythrocytes (T) (b) and schizont-infected erythrocytes (S) (c). Each panel contains from left to right: bright field, DAPI-stained nuclei, anti-Pf322, anti-SBP1 and overlay of all four. Free merozoites (M) are shown in d. d is as above except that the fourth panel was labelled with anti-MSP1 antibodies. Panel row e contains free merozoites and material positive for both Pf332 and SBP1 suggesting Maurer's clefts (white arrows) that were released during schizont rupture. Panel row f shows CS2ΔPf322 schizont-infected erythrocytes (S). The order of each panel is as described above. B. Transmission electron microscopy performed on high pressure frozen and freeze substituted sections of CS2-infected erythrocytes. The sections were labelled with either rabbit or mouse anti-Pf332 antibody and show the distribution of Pf332 in the red blood cell cytosol (a and b) and on Maurer's clefts (c and inset). C. Live IFA on CS2-infected erythrocytes to determine whether Pf332 is on the surface of merozoites and/or trophozoite-infected erythrocytes. The first column of each row shows a bright field image, the second column a nuclear stain (DAPI), the third column reactivity with mouse monoclonal anti-Pf332 antibody and the last column an overlay of the previous three columns. Panels a and b: reactivity of anti-Pf332 antibodies with free live merozoites (M) and trophozoite (T)-infected erythrocytes to test for surface localization. Panel c: control panel with free live merozoites labelled with anti-MSP1 antibodies. Panel d: control panel with live trophozoites labelled with human serum (HS) from an individual living in a malaria-endemic region. Panel e: control panel with anti-PfEMP3 antibodies with live trophozoite-infected erythrocytes. Panel f: IFA on trophozoite-infected erythrocytes permeabilized with Triton X-100 to prove reactivity of the mouse monoclonal anti-Pf332 antibody.

To determine the subcellular localization of Pf332 parasites were labelled using a specific monoclonal antibody and colocalized with skeleton-binding protein 1 (SBP1), a known Maurer's clefts marker in the *P. falciparum*-infected erythrocyte (Blisnick *et al*., 2000). The Pf332 protein could not be detected in ring stages (Fig. 6A, a); however, it was strongly expressed in trophozoite and schizont stages showing colocalization with SBP1 in Maurer's clefts consistent with previous subcellular localization of this protein (Fig. 6A, b and c) (Moll *et al*., 2007). The truncated Pf332 protein in CS2Pf332trunc showed the same subcellular localization in Maurer's clefts as the wild-type protein showing that the necessary signals for correct trafficking were contained within this 87 kDa region (data not shown). No expression of Pf332 could be detected in free merozoites (Fig. 6A, d and e); however, material that resembled intact Maurer's clefts was located outside schizont stages suggesting that they were released on rupture of the cell (Fig. 6A, e). Both Pf332 and SBP1 localized to these structures consistent with release of Maurer's clefts on erythrocyte rupture (Fig. 6A, e, white arrows). Expression of Pf332 was, as expected, absent in CS2ΔPf332 (Fig. 6A, f). Similar subcellular localization results and Pf332 detection were obtained using the rabbit polyclonal anti-Pf332 antibodies (data not shown).

An interesting feature that became evident with colocalization of Pf332 and SBP1 to Maurer's clefts was that the former protein was not present in these structures in ring stages, as this protein is expressed later in the blood-stage life cycle. Therefore Maurer's clefts are released from the parasitophorous vacuole in ring stages with cargo such as SBP1 before Pf332 is expressed. Maurer's clefts formed later in the life cycle after expression of Pf332 contain this protein. Therefore there is a mixed population of Maurer's clefts structures that are formed from the parasitophorous vacuole through ring and trophozoite stages that contain different protein cargoes (Fig. 6A, a and b).

Confirmation of Pf332 localization to Maurer's clefts was obtained using immunoelectron microscopy in which consistent labelling was obtained on these structures with some specific labelling of electron-dense material within the *P. falciparum*-infected erythrocyte (Fig. 6B). Labelling could also be observed in the erythrocyte cytoplasm and the parasitophorous vacuole membrane (Fig. 6B).

### The Pf332 DBL domain does not bind to erythrocytes or play a role in merozoite invasion

The Pf332 DBL domain has been previously expressed as a glutathione-S-transferase fusion protein and reported to interact with the erythrocyte surface (Blisnick *et al*., 2000). We expressed a similar DBL region as a single refolded domain and used this protein in erythrocyte binding assays. However, no binding of erythrocytes could be detected in multiple experiments using different batches of the DBL domain (data not shown). While we have shown that Pf332 is not expressed in merozoites we investigated the possibility that this protein was released into the supernatant, which would potentially allow it to interact with the erythrocyte and indirectly play a role in invasion. Immunoblots of parasite supernatants after schizont release were analysed to detect any soluble Pf332 DBL domain. It was not possible to detect this domain in supernatant from these ruptured erythrocytes (Fig. S3) indicating the protein was retained in the insoluble membranous material and suggesting furthermore that it is not released from Maurer's clefts (data not shown). This is consistent with the presence of seemingly intact Maurer's clefts outside parasite-infected erythrocytes as shown by immunofluorescence (Fig. 6A, e). Additionally, we found that the antibodies to the refolded Pf332 DBL domain were not able to inhibit merozoite invasion in growth inhibition experiments (data not shown). Combined, the above data are consistent with Pf332 not playing a role in merozoite invasion nor is the protein present in a localization consistent with any role in this parasite function.

### The DBL domain of Pf332 is not exposed on the surface of CS2-infected erythrocytes

**Fig. 7:**
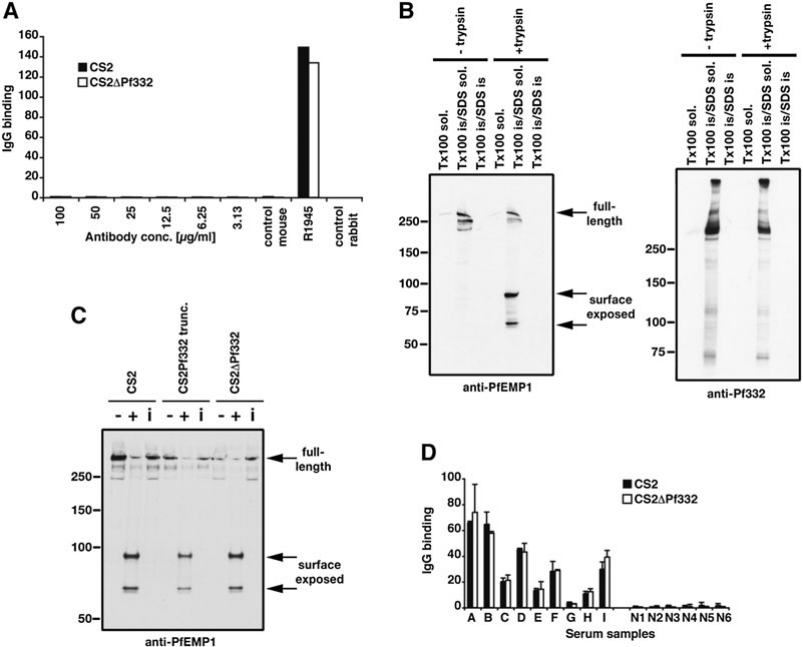
Pf332 is not located on the surface of *P. falciparum*-infected erythrocytes and not required for surface localization of PfEMP1. A. FACS analysis of *P. falciparum*-infected trophozoites to test for the presence of Pf332 on the surface with increasing amounts of anti-Pf332 antibodies. The control sera R1945 was made by repeated injection of live CS2 parasites into rabbits. B. Trypsin treatment of CS2-infected erythrocytes to determine surface localization for Pf332 compared with PfEMP1. The first panel contains parasites either treated or not treated with trypsin and differentially solubilized to show that PfEMP1 is sensitive to trypsin and contained within the Triton X-100-insoluble/SDS-soluble fraction. The second panel shows the same samples probed with anti-Pf332 antibodies to show that the protein is resistant to trypsin in intact cells and that it is also within the Triton X-100-insoluble/SDS-soluble fraction. C. Trypsin treatment of intact erythrocytes infected with CS2, CS2Pf332trunc and CS2ΔPf332 to determine the presence of PfEMP1 on the surface of the host erythrocyte. For each cell line three lanes are shown: treatment without (−) and with (+) trypsin and treatment with trypsin in the presence of trypsin inhibitor (i). Full-length PfEMP1 and its cytoplasmic tail were detected using antibodies to the cytoplasmic acidic terminal segment (ATS). Full-length PfEMP1 was detected as a > 300 kDa band. Surface exposed PfEMP1 was cleaved by trypsin and results in the detection of bands between 70 and 90 kDa. D. IgG binding to the surface of CS2- and CS2ΔPf332-infected erythrocytes using sera from malaria-exposed multigravid women from Papua New Guinea (samples A–I) compared with sera from Melbourne residents as non-exposed controls (Samples N1–5). Values are means (+SEM) from three experiments in duplicate.

Previously, Pf332 was detected on the *P. falciparum*-infected erythrocyte surface by immunofluorescence with polyclonal antibodies to a recombinant DBL domain for the strains FCR3 and HB3 (Moll *et al*., 2007). Using live CS2ΔPf332-infected erythrocytes lacking expression of Pf332 as a negative control we tested whether this protein was exposed on the host cell surface with the monoclonal antibody in immunofluorescence experiments (Beeson *et al*., 2006). The CS2 cloned line is derived from ItG2 and is essentially the same parasite line as most laboratory FCR3 strains due to a contamination event before distribution of these parasites around the world (Rubio *et al*., 1995). No reactivity with the monoclonal antibody was observed for CS2 with either free merozoites or trophozoite stages suggesting that Pf332 was located neither on the surface of the parasite invasive stage nor on the infected erythrocyte (Fig. 6C, a and b), which is in contrast to previous results (Moll *et al*., 2007). Similar results were observed for the strains 3D7 and HB3 (data not shown). As positive controls we used antibodies to MSP1 and human serum from an individual living in a malaria-endemic region. As expected the MSP1 antibodies and the human serum reacted with the surface of the merozoite and trophozoite-infected erythrocyte respectively (Fig. 6C, c and d) (Holder and Freeman, 1982). A monoclonal antibody to PfEMP3, a protein located underneath the *P. falciparum*-infected erythrocyte membrane, was used as negative control and as expected showed no reactivity (Waterkeyn *et al*., 2000) (Fig. 6C, e). The monoclonal antibody against Pf332 only gave specific fluorescence when the erythrocyte membrane was permeabilized indicating that this antibody could detect the DBL domain of this protein in each of the strains tested but not in live and intact parasites or parasite-infected erythrocytes (Fig. 6C, f). FACS analysis with increasing concentrations of antibody confirmed this result as Pf332 was not detectable on live parasite-infected erythrocytes (Fig. 7A). These results are consistent with Pf332 not being located on the CS2, HB3 and 3D7 *P. falciparum*-infected erythrocyte surface.

To further confirm that Pf332 was absent from the surface of *P. falciparum*-infected erythrocytes we used an assay developed for detecting surface expression of PfEMP1 (Waterkeyn *et al*., 2000). Trypsin treatment of CS2-infected erythrocytes results in digestion of the surface PfEMP1 ectodomain leaving the cytoplasmic acidic terminal sequence (ATS), which can be detected with antibodies raised to the C-terminus of the protein (Fig. 7B). The PfEMP1 protein and the residual cytoplasmic region remaining after tryptic digestion were Triton X-100 insoluble and SDS soluble as was the Pf332 protein. Identically treated samples were probed with anti-Pf332 antibodies that react with the large repetitive cytoplasmic domain. *P. falciparum*-infected erythrocytes, which were treated with and without trypsin, gave an identical band pattern on immunoblots, with the dominant Pf332 bands visible at approximately 700 and 400 kDa (Fig. 7B). This outcome is consistent with Pf332 not being exposed on the surface of *P. falciparum*-infected erythrocytes and remaining localized to Maurer's clefts. Moreover, it shows that PfEMP1 and Pf332 share the same solubility characteristics.

### PfEMP1 export is not affected in CS2ΔPf332-infected erythrocytes

Pf332 is expressed and trafficked to Maurer's clefts when PfEMP1 begins to appear on the erythrocyte surface suggesting it may play a role in transfer of this virulence protein to the erythrocyte surface (Waterkeyn *et al*., 2000). To investigate whether trafficking and display of PfEMP1 is affected by the truncation or deletion of Pf332, we performed trypsin cleavage assays to detect the surface pool of this protein (Fig. 7C) (Waterkeyn *et al*., 2000). As above, cultures were treated with trypsin, and PfEMP1 protein detected using antibodies to the C-terminal conserved ATS, a region located on the cytoplasmic side of the infected erythrocyte membrane. In CS2 a large protein band of over 300 kDa was detected by anti-ATS antibody, representing the full-length protein. In the absence of trypsin, or when the action of trypsin was inhibited by soybean trypsin inhibitor, only full-length PfEMP1 was observed, whereas the addition of trypsin leads to the detection of products of 70 and 90 kDa. These bands represent the protected intracellular PfEMP1 domain (ATS and transmembrane region) after cleavage of surface-exposed PfEMP1. Full-length PfEMP1 can also be detected in trypsin treated cells, which represents the non-exposed PfEMP1 pool in these cells. In the three cell lines, CS2, CS2Pf332trunc and CS2ΔPf332, PfEMP1 was clearly located on the surface. CS2- and CS2ΔPf332-infected erythrocytes had approximately the same levels of surface PfEMP1 indicating correct trafficking and exposure of this protein independent of Pf332 function. However, the CS2Pf322trunc-infected erythrocytes appeared to have a decreased level and this may be due to a dominant-negative effect as was seen previously when PfEMP3 was truncated (Waterkeyn *et al*., 2000). Truncation of PfEMP3 resulted in partial blockage of PfEMP1 so that decreased levels were transferred to the infected erythrocyte surface. When a parasite lacking expression of the full PfEMP3 protein was constructed this effect disappeared suggesting the blockage was physical rather than a direct effect on function (i.e. a dominant-negative effect) (Waterkeyn *et al*., 2000).

The presence of similar amounts of PfEMP1 on the surface of CS2 and CS2ΔPf332 was confirmed by measuring the binding of IgG from malaria-exposed individuals to infected erythrocytes of CS2 and CS2ΔPf332 (Beeson *et al*., 2006). Using serum from malaria-exposed multigravid women from Papua New Guinea, which reacts predominantly with the PfEMP1 protein encoded by var2csa, IgG binding to the surface of infected erythrocytes of CS2 and CS2ΔPf332 was very similar (Fig. 7D). Furthermore, rabbit antibodies raised against CS2-infected erythrocytes recognize PfEMP1 (Elliott *et al*., 2005), labelled CS2 and CS2ΔPf332 at similar levels (R1945; Fig. 7A).

### Truncation of Pf332 alters P. falciparum-infected erythrocyte deformability

**Fig. 8:**
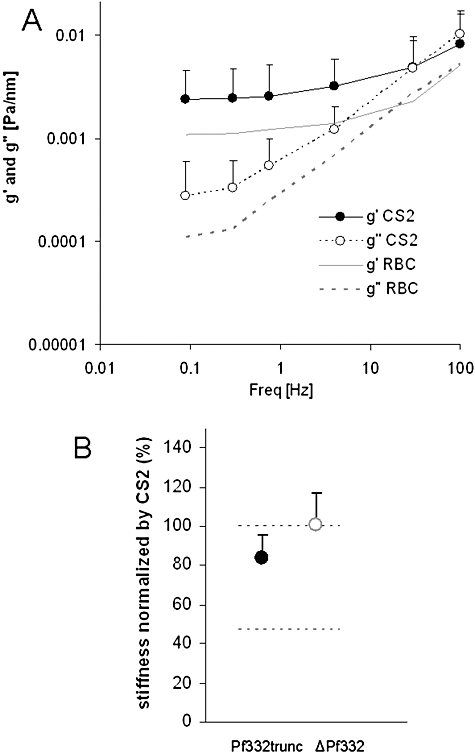
A. Effect of *P. falciparum* on erythrocyte deformability: stiffness, g′ (closed data symbols), and friction, g″ (open data symbols), of CS2 infected erythrocytes, measured at frequencies from 0.1 to 100 Hz, CS2 (*n* = 16). Stiffness (grey continuous line) and friction (grey dashed line) of uninfected erythrocytes. Data points are geometric means and SD. B. Stiffness data of CS2Pf332trunc- (*n* = 18) and CS2ΔPf332- (*n* = 15) infected erythrocytes, normalized by the parental line CS2. All data are geometric means and SE, and are taken at 0.75 Hz. Black dashed line at 100% represents CS2 and grey dashed line is the stiffness of the uninfected erythrocytes.

To assess if Pf332 plays a role in determining erythrocyte rigidity we used optical magnetic twisting cytometry (Puig-de-Morales-Marinkovic *et al*., 2007). This technique involves small deformations localized mainly to the vicinity of the bead. Therefore these measurements reflect primarily the contribution from the cell membrane, which is mainly determined by the erythrocyte cytoskeleton (Puig-de-Morales-Marinkovic *et al*., 2007). First, we examined the loss of deformability of the erythrocyte due to the presence of the parental CS2 at trophozoite stages. Second, we examined the role of Pf332 in membrane deformability by comparing CS2Pf332trunc, which has a greatly truncated C-terminal tail, with CS2ΔPf322 lacking the expression of this large protein. The storage modulus or stiffness, g′, for the CS2-infected erythrocytes (Fig. 8A, closed data points) shown almost no increase with increasing frequency. The loss modulus or friction, g″ (Fig. 8A, open data points) was approximately an order of magnitude smaller than g′, which indicates that the elastic component clearly dominates. Friction showed a much stronger dependence upon frequency than did stiffness. These trends are comparable to those reported previously (Puig-de-Morales-Marinkovic *et al*., 2007). When compared with uninfected erythrocytes (Fig. 8, grey line), we found that the presence of the parasite stiffens the cell membrane by more than twofold. Regardless of the parasite, frequency dependencies of g′ and g′′ were similar to those seen with the parental line (data not shown). Therefore, to quantify the effect of Pf332 truncation or null expression we first focused upon responses of g′ measured at 0.75 Hz. For the specific purpose of comparison between CS2Pf332trunc and CS2ΔPf332, normalized means are plotted with standard error (Fig. 8B, data points). We have also included the median stiffness value of uninfected cells (47%). While the median stiffness of the CSPf332trunc-infected erythrocytes is ∼85%, representing ∼15% decrease, the more appropriate comparison is the change in stiffness relative to that associated with infection. In particular, this shows that ∼30% of the difference in membrane hardening due to the presence of the parasite is related to the truncation of Pf332.

We checked for significance between the three groups (CS2, CS2Pf332trunc and CS2ΔPf332) at that particular frequency and found that there was little change in g′ for CS2ΔPf332 compared with CS2 but a significant decrease for CS2Pf332trunc compared with CS2 (*P*< 0.05). We then extended the comparison to the entire frequency range (see *Experimental procedures*) and we corroborated our findings. The comparison between CS2- and CS2Pf332trunc-infected erythrocytes showed statistical significance (*P*< 0.0005) while the comparison between CS2 and CS2ΔPf332 did not.

## Discussion

Following merozoite invasion *P. falciparum* initiates a remarkable remodelling process making the host erythrocyte more rigid and able to adhere to receptors (see for review Marti *et al*., 2005). This involves export of proteins that includes Pf332, a large protein that contains a DBL region, a domain that occurs only within *Plasmodia* spp. (Mattei and Scherf, 1992; Gardner *et al*., 2002). In order to better understand the role of this domain and the Pf332 protein we expressed a functional form to assess its similarity to the known structures for other DBL domains. The recombinant DBL domain of Pf332 had physicochemical properties and a disulphide-bonding pattern consistent with a correctly refolded protein. In comparison with the parental disulphide-bond pattern found in the F2 domain of EBA-175, a disulphide bond from both subregions I and III were found to have been lost (Tolia *et al*., 2005). An additional disulphide bond (Cys-5a–Cys-6a) was also found in subregion II and formed a surface ridge within the area equivalent to the hydrophobic pocket of the DARC binding site in Pk-αDBL (Singh *et al*., 2006). This disulphide bond was validated experimentally and could easily be accommodated in the modelled structure for the Pf332 DBL domain. These observations provide strong evidence that the recombinant Pf332 DBL domain represents a functional domain.

Although the Pf332 DBL domain has its greatest homology to the F2 domain (28% amino acid identity) of EBA-175, it does not possess the two adjacent DBL domains found in the R2 region that is a characteristic of the ebl family. Hence Pf332 structure is not consistent with the dimerization mechanism of binding proposed for EBA-175 with an F1/F2 domain interaction from two opposing molecules (Tolia *et al*., 2005). The construction of the Pf332 molecule is most similar to that found for the more ancient Duffy-binding proteins found in various *Plasmodium* spp. These proteins are type 1 membrane-spanning proteins which are comprised of: (i) an N-terminal, single DBL domain which binds DARC on erythrocytes, (ii) a centrally located cysteine-depleted region, (iii) a small cysteine-rich domain adjacent to the membrane-spanning region, and (iv) a small C-terminal cytoplasmic tail. Pf332 does not have the small cysteine-rich domain adjacent to the transmembrane-spanning domain and has an extremely large cytoplasmic tail (> 600 kDa). Comparison between the modelled structure for Pf332 DBL with that of Pkα-DBL revealed that most of the important features and residues within this site were not present in the equivalent region of the Pf332 DBL domain. Hence the structural features of the Pf332 DBL domain are inconsistent with involvement in erythrocyte binding via the current documented mechanisms (Tolia *et al*., 2005; Singh *et al*., 2006). This is consistent with our inability to demonstrate binding of the Pf332 DBL domain to erythrocytes, absence of the protein on the red blood cell surface and lack of release of a soluble form of this domain into the supernatant after parasite egress.

Previous data have suggested that Pf322 is located on the surface of the host-infected erythrocyte and that it plays a role in merozoite invasion of erythrocytes perhaps by initial binding of the DBL domain on the schizont-infected host cell surface with uninfected erythrocytes (Mattei and Scherf, 1992; Moll *et al*., 2007). Earlier studies reported that antibodies raised to the large repetitive region of Pf332 could inhibit invasion but this was most likely due to the cross-reactive nature of these antibodies rather than specific blocking of protein function (Ahlborg *et al*., 1995). More recent data have suggested that antibodies raised to a GST–Pf332 DBL fusion protein could give at best a very modest inhibition of merozoite invasion (Moll *et al*., 2007). The Pf322 DBL domain expressed in our studies did not show any binding to red blood cells in contrast to previous data where this domain was made as a GST chimeric protein and also expressed in CHO cells (Moll *et al*., 2007). However, the localization of Pf332 is relevant in providing clues with respect to the possible function of this protein. We have shown that the Pf332 DBL domain is not present on the surface of *P. falciparum*-infected erythrocytes. The protein remains within Maurer's clefts and is released during merozoite egress associated with insoluble membranous material. Pf332 is therefore not available to interact with uninfected erythrocytes and is also not associated with free merozoites so that its subcellular localization is inconsistent with it playing any role in invasion. This is consistent with lack of invasion inhibition using antibodies against the correctly folded DBL domain and also lack of Pf332 DBL domain binding to erythrocytes. Also the Pf332 DBL domain shows essentially no sequence diversity in different parasite strains indicating no selective pressure by the immune system. This is in contrast with the EBA-175 F1/F2 region, an important invasion ligand that shows considerable diversity as a result of strong selective pressure (Baum *et al*., 2003). This is consistent with the lack of exposure of the Pf322 DBL domain on the erythrocyte or merozoite surface and its lack of involvement in invasion.

As Pf332 remains within Maurer's clefts in the *P. falciparum*-infected erythrocyte it is likely that it serves a function within the trophozoite and schizont stages. The most likely possibilities were, first, that it is involved in trafficking the cytoadherence protein PfEMP1 to the host cell surface and/or, second, that it contributes to the rigidification of the parasite-infected erythrocyte. PfEMP1 trafficking was not affected in Pf332 null parasites demonstrating that it was not required for this function at least in this parasite strain. Nevertheless, we cannot rule out involvement of this protein in PfEMP1 trafficking, as it is possible that there is functional redundancy and other proteins play this role in the absence of Pf332 (Maier *et al*., 2008).

The truncation of Pf332 shows a significant effect on the stiffness of the *P. falciparum*-infected erythrocyte. Because of the measurement technique, these stiffness measurements reflect mainly the contribution from the cell membrane and closer vicinities rather than from other internal sources (Puig-de-Morales-Marinkovic *et al*., 2007). Hence, the effect of the truncation on membrane stiffness, which may be a dominant-negative effect, is consistent with a role for the large cytoplasmic tail of Pf332 interacting with the already remodelled membrane cytoskeleton. The stiffness data and protein location combined suggest that Pf332 may contribute to the rigidity of the parasite-infected host and in particular Pf332 DBL domain may be involved in anchoring and scaffolding. While this work was in review a similar study was published showing that loss of Pf332 expression resulted in increased rigidity of the *P. falciparum-*infected erythrocyte consistent with the results observed here (Glenister *et al*., 2008). Additionally, they found that PfEMP1 trafficking was decreased resulting in less of the adherence protein on the erythrocyte surface. There are two possible reasons for the differences observed with our work. First, we have used the strain CS2 that expresses higher levels of PfEMP1 while they used 3D7 and it is possible the disparate results could be explained by expression of different redundant genes involved in the same process. Second, it is possible that they are observing a dominant negative effect in which truncated Pf332 is interfering with trafficking of PfEMP1 and that it does not act directly in this process. We strongly favour the latter explanation and this would be similar to a previous study by us in which a truncated PfEMP3 protein caused decreased PfEMP1 trafficking to the erythrocyte surface by physical blockage rather than the full-length protein playing any essential role in the process (Waterkeyn *et al*., 2000).

Unexpectedly, the complete removal of Pf332 function had no effect on membrane stiffness. Given the results with the truncated protein, a plausible explanation is that there is protein redundancy. In the case of the CS2Δf332 parasite the full deletion of Pf332 does not affect erythrocyte rigidity suggesting that there are many parasite proteins involved in determining this phenotype, which may overlap in parasite function. Indeed, we have recently identified other proteins that may play a role in determining the overall rigidity of the *P. falciparum*-infected erythrocyte (Maier *et al*., 2008). However, in the case of the CS2Pf332trunc parasite Pf332 is still present although truncated, and consequently occupying the place of other proteins. This idea is also supported by the fact that there is a small reduction in PfEMP1 trafficking in the parasites expressing the truncated protein but not the knockout parasites. Taken together these results indicate that other proteins are also involved in the loss of deformability and that the parasite may use redundancy as a strategy.

The experiments presented here have defined the disulphide bonds of the Pf332 DBL domain suggesting that there is similarity with those of Pkα-DBL and EBA-175 (Tolia *et al*., 2005; Singh *et al*., 2006). While the DBL domain is *Plasmodium* spp. specific it is present in a large number of proteins with differing functions. The DBL domain is important in binding to proteins and it is possible that in Pf332 this domain interacts with proteins in Maurer's cleft.

## Experimental procedures

### Cloning, expression and purification

The Pf332 DBL domain including residues S10 to A262 was inserted into the expression vector pProExHTb (Invitrogen, Carlsbad, CA, USA) with an N-terminal hexa-His tag. It was produced and purified in a manner similar to that described previously (Hodder *et al*., 2003). Briefly, the protein was synthesized in *E. coli* strain BL21(DE3) for 3 h at 37°C and deposited in insoluble inclusion bodies. The cells were lysed by sonication and the insoluble inclusion bodies solubilized by the addition of 6 M guanidine-HCl, pH 8.0. The solubilized protein was isolated by metal-chelate chromatography using Ni-NTA agarose resin (Qiagen, Hilden, Germany), and eluted in 8 M urea, 1 M imidazole, pH 8.0. The protein was diluted 100-fold and refolded at room temperature for 24 h in 2 M urea, 100 mM NaCl, 20 mM Tris pH 8.0, with 1 mM reduced and 1 mM oxidized glutathione included to facilitate disulphide bond formation. One tablet of Complete Protease inhibitors (Roche, Basel, Switzerland) was added per 200 ml of refold solution to prevent unwanted degradation of Pf332 in the refold mixture. Refolded protein was isolated by anion-exchange chromatography and fractions containing monomer were pooled for further use.

### RP-HPLC analysis of the refolding reaction

RP-HPLC was performed using an Agilant 1100 modular HPLC consisting of an on-line degasser, piston pump, autosampler, column oven, diode-array detector and fraction collector. Instrument control, data acquisition and evaluation were performed using Hewlett–Packard Chemstation software for LC and LC/MS systems. Buffer A was comprised 0.05% (v/v) trifluoroacetic acid (HPLC/Spectro grade, Pierce, Rockford, IL, USA) in Milli-Q grade water (Millipore, Bedford, MA, USA), while buffer B was comprised of 0.05% (v/v) trifluoroacetic acid in acetonitrile (ChromAR HPLC grade, Malinckrodt, Paris, KY, USA). Aliquots from the refold mixture were centrifuged at 14000 r.p.m. for 15 min at room temperature prior to loading onto a C8 column [2.1 mm inner diameter × 100 mm (Brownlee columns, Perkin-Elmer Instruments, Norwalk, CT, USA)] in the presence of buffer A. Bound proteins were eluted using a linear gradient of 0–100% buffer B over 12 min at a flow rate of 0.5 ml min^−1^ at 37°C.

### Production of antibodies

Polyclonal and monoclonal antibodies against the refolded recombinant antigen were generated in rabbits and mice, with each animal receiving a minimum of three immunizations prior to assessing the reactivity of the antibodies to the parasite antigen on immunoblots and immunofluorescence assays.

### SDS-PAGE and immunoblots procedures

Parasite cultures were synchronized by treatments with sorbitol (Lambros and Vanderberg, 1979), and in some cases enriched by percoll purification (Aley *et al*., 1986). Parasite-derived proteins were fractionated on Tris-Acetate 3–8% precast gels (Invitrogen, Carlsbad, CA, USA) using the manufacturer's running buffer. For analysis of the high-molecular-weight forms of Pf332 found in parasites, electrophoresis was continued until the 50 kDa marker reached the bottom of the gels. Recombinant proteins were fractionated on 4–12% Bis-Tris precast gels (Invitrogen, Carlsbad, CA, USA) using MES running buffer. Proteins were electropherically transferred to PVDF membrane (0.22 μm, Millipore, Billerica, MA, USA). Immunoblotting was performed using procedures described previously (Hodder *et al*., 2001). The following antibodies were used: mouse anti-Pf332 serum (1:500), mouse anti-Pf332 mAb 10H2 (10 μg ml^−1^), mouse anti-ATS (1B98 6HI) (1:100) (pre-absorbed on erythrocyte ghosts) and mouse anti-HSP70 (1:2500). Horseradish peroxidase-coupled sheep anti-mouse Ig (1:2000, Chemicon, Melbourne, Australia) was used as a secondary antibody. The protein bands on the immunoblots were visualized using a chemiluminescent substrate (Pierce, Rockford, IL, USA) according to the manufacturer's instructions.

### Tryptic digestion of refolded Pf332 DBL domain

RP-HPLC purified Pf332 DBL domain (approximately 6 nmol) was reduced to half volume using a Centrivap concentrator (Labconco, Fort Scott, KS, USA). The solution was then buffered to pH 6.5 using a 1:10 dilution of 1 M sodium phosphate, pH 6.5. Proteomics grade trypsin (Sigma, St Louis, MO, USA) in 1 mM HCl was added at an enzyme:protein ratio of 1:25 (w/w), and the solution was incubated at 37°C for 4 h. A second aliquot of trypsin (1:25 w/w) was then added, and the mixture incubated for a further 16 h at 37°C. Digestion was stopped either by snap freezing to −70°C or RP-HPLC.

### RP-HPLC determination and fractionation of disulphide-bonded tryptic peptides from Pf332 DBL domain

Analytical RP-HPLC of either DTT-reduced or non-reduced Pf332 tryptic digests was used to identify the location of disulphide-linked peptides within the elution profile in a manner similar to that described previously (Hodder *et al*., 1996). RP-HPLC was conducted using the Agilant 1100 system and buffers described above. An aliquot containing 20 μg of tryptic digest was mixed with an equal volume of 6 M guanidinium chloride, 0.2 M Tris-HCl, pH 8.4, 5 mM EDTA and then reduced by the addition of DTT (final concentration, 25 mM) for 60 min at 45°C. A tryptic fingerprint for the reduced digest was then obtained by eluting the peptide fragments from a C18 column (4.6 mm inner diameter × 250 mm, Vydac, Grace Davison, Hesperia, CA, USA) using a 0–70% buffer B linear gradient over 120 min at 45°C and a flow rate of 1 ml min^−1^. The profile obtained was then compared with the tryptic fingerprint obtained for a similar amount of non-reduced digest eluted under identical chromatographic conditions. The disappearance of a peak following reduction indicated the presence of a disulphide-linked peptide. The remainder of the digest mixture (125 μg) was then fractionated under identical conditions. The disulphide-containing peptides were collected and subjected to Edman degradation and ion-trap mass-spectrometric analysis.

### Edman degradation and mass spectrometric analyses of tryptic peptides

RP-HPLC fractions were subjected to direct amino acid sequence analysis (Edman degradation) with pulsed-liquid delivery of critical reagents in an automated sequenator (Model 492, Applied Biosystems, Foster City, CA, USA). Individual fractions were infused directly into the nanospray ion source of the a LCQ Classic quadrupole ion-trap mass spectrometer (Thermo) with the following settings: nanospray tip voltage 2.3 kV, capillary temperature 150°C, capillary voltage 24 V, tube lens offset 5 V. The AspN subdigestion of tryptic fragment #35 (approximately 1 μg as determined by Edman degradation results) was done as per manufacturer's recommended procedure (Roche, Basel, Switzerland) using AspN sequencing grade enzyme in 50 mM sodium phosphate, pH 8.0 solution at an enzyme:peptide ration of 1:20 for 2 h at 37°C. The subdigest of tryptic fraction #35 was analysed after clean-up on C18 ZipTip (Millipore, Bedford, MA, USA) by RP-HPLC on a C18 column (3 μm, 0.15 mm inner diameter × 150 mm, Vydac) using a Surveyor-MS HPLC system (Thermo, San Jose, CA, USA). Mobile-phase buffers were 0.2% (v/v) formic acid in water (buffer A) and 0.2% (v/v) formic acid in acetonitrile (buffer B), and were run at a flow rate of 1 μl min^−1^ with a linear gradient of 5–95% buffer B over 20 min, held at 95% buffer B for 10 min and finally 95–5% buffer B over 2 min. The effluent from the column was connected directly to the nanospray ion source of the LCQ MS. MS-Bridge software (http://prospector.ucsf.edu/cgi-bin/msform.cgi?form=msbridgestandard) was used to identify molecular ions corresponding to the expected theoretical cleavage products of tryptic fraction #35.

### Modelling of the Pf332 DBL domain

The model of Pf332 was created with the modeller (6v2) program (Fiser and Sali, 2003) using the X-ray crystal structure of EBA-175 (domain 2) (Tolia *et al*., 2005) (PDB access code 1ZRO) as a template, and the sequence alignment shown in Fig. S1. From 25 initial models the structure with lowest Modeller Objective Function was subjected to molecular dynamics (MD) simulation. All MD calculations were performed using the gromacs (v3.1.4) package of programs (Lindahl, 2001) with the OPLS-aa force field (Smith *et al*., 1998). The protein was placed in a 90 × 90 × 90 Å^3^ water box with no pressure coupling. Ionizable residues were assumed to be in their standard state at neutral pH – the total charge on the system was made neutral by replacing water molecules with sodium ions. The LINCS algorithm (Hess *et al*., 1997) was used to constrain bond lengths. Peptide, water and ions were coupled separately to a thermal bath at 300 K using a Berendsen thermostat (Berendsen *et al*., 1984) applied with a coupling time of 0.1 ps. All simulations were applied with a single non-bonded cut-off of 10 Å, using a neighbour-list update frequency of 10 steps (20 fs). The particle-mesh Ewald method was applied to deal with long-range electrostatics – a grid width of 1.2 Å was used with a forth-order spline interpolation. All simulations consisted of an initial minimization of water molecules followed by 10 ps of MD with the protein fixed. Following positional restraints MD, the restraints on the protein were removed and MD continued for a further 1 ns. The time-step used in all MD simulations was 2 fs. At the completion of the MD calculation the system was minimized using the method of steepest descents.

### Plasmid construction, parasite strains, culture conditions and transfection

A deletion construct was generated by insertion of a 5′[619 base pairs (bp)] and 3′ (512 bp) segment of Pf332 into pCC-1 (Maier *et al*., 2008), with SacII/SpeI and EcoRI/NcoI. The segments were amplified from CS2 genomic DNA using oligonucleotide primers aw814/aw815 and aw816/aw817 respectively. The truncation plasmid was derived from pHHT-TK (Maier *et al*., 2006) by insertion of a 5′ flank (901 bp, amplified by aw152/153) and a 3′ flank (939 bp, amplified by aw154/155) using SacII/SpeI and EcoRI/AvrII as restriction enzymes.

*Plasmodium falciparum* asexual stages of the CS2 clone (Duffy *et al*., 2006) were maintained in human 0+ erythrocyte. Transfection with 100 μg of purified plasmid DNA (Qiagen, Hilden, Germany and Invitrogen, Carlsbad, CA, USA) and selection for stable transfectants were carried out as described (Maier *et al*., 2006; 2008).

### Oligonucleotides and DNA analysis

The following oligonucleotides were used:

Aw152: atcccgcggaagaggatgtgcaggaattag,
Aw153: gatactagtctactggtaactcttctatgggac,
Aw154: atcgaattcggatcagtcactgaacaacttgttg,
Aw155: gatcctaggcacctttagtgtgtatatcagcagtag,
Aw814: atcccgcggtgtagaacttatgctg,
Aw815: gatactagtctctcatcatgcttctcacg,
Aw816: ggtgagagtattatggatagaattcc,
Aw817: ggatccatggtttctaattcctgcacatcctc.

Restriction sites introduced for cloning purposes are underlined. Genomic DNA was prepared with the DNeasy Tissue Kit (Qiagen) and subjected to Southern Blot analysis using the Roche DIG system following the manufacturer's instructions.

### Trypsin cleavage assay

Parasites were synchronized by sorbitol and enriched by gelofusin floatation (Braun, Melsungen, Germany) at the early trophozoite stage. The cells were then treated with either TPCK-treated trypsin (Sigma) 1 mg ml^−1^ in PBS or TPCK-treated trypsin plus soybean trypsin inhibitor (5 mg ml^−1^ in PBS, Worthington, Lakewood, NJ, USA) or PBS alone for 1 h at 37°C. After incubation soybean trypsin inhibitor was added followed by incubation at room temperature for 15 min. Cell pellets were lysed with Triton X-100 (1%), and insoluble proteins were collected and re-suspended in SDS (2%) as described (Waterkeyn *et al*., 2000) before use in Western blotting.

### Immunofluorescence and transmission electron microscopy

For immunofluoresence analysis, either acetone/methanol (90%/10%) fixed smears or live cells of asynchronous parasites of CS2ΔPf332-, CS2truncPf332- and CS2-infected erythrocytes were used. After washing in PBS and blocking in 0.5% BSA the samples were probed with either polyclonal rabbit or monoclonal mouse anti-Pf332 (1:500), rabbit anti-SBP1 (1:500), rabbit anti-MSP1 (1:200), rabbit anti-ATS (1:200), rabbit anti-PfEMP3 (1: 2000) or with human serum from a malaria-endemic area in Kenya (1:20) and consequently incubated with secondary antibodies Alexa-Fluor 488-conjugated anti-rabbit IgG (Molecular Probes) and Alexa-Fluor 594-conjugated anti-mouse IgG (Molecular Probes). Cells were viewed with an Apochromat 100×/1.4 oil lense on a Zeiss Axioskop 2 microscope equipped with a PCO SensiCam (12bit) camera and Axiovision 3 software. Captured images were processed using Photoshop and ImageJ software (available from http://rsb.info.nih.gov/ij). Pictures were adjusted to gain optimal contrast to visualize features of interest. For transmission electron microscopy wild-type CS2-infected erythrocytes were magnet-purified at the trophozoite stage and high pressure frozen, freeze substituted and processed further as described earlier (Rug *et al*., 2004). Ultra-thin sections were probed with polyclonal rabbit or monoclonal mouse anti-Pf332 (1:100), followed by gold-conjugated anti-rabbit (20 nm) or anti-mouse (10 nm) IgG, stained additionally for contrast as described earlier (Rug *et al*., 2004) and subsequently viewed under a Philips CM transmission EM at 120 kV.

### Flow cytometry

Binding of antibodies (human, rabbit and monoclonal) to the surface of *P. falciparum*-infected erythrocytes was measured by flow cytometry using established methods (Beeson *et al*., 2004). *P. falciparum* mature trophozoite stage-infected erythrocytes were tested at 2–4% parasitaemia and 0.2% haematocrit. Where infected erythrocytes were tested for binding of antibodies in human sera, cells were sequentially incubated with test serum (1/20), rabbit anti-human IgG (Dako) and Alexa-Fluor 488-conjugated anti-rabbit IgG (Molecular Probes, 1:1000), with ethidium bromide (10 μg ml^−1^) in darkness. When using rabbit antibodies (R1945), cells were sequentially incubated with rabbit antiserum (1/25) and Alexa-Fluor 488-conjugated anti-rabbit IgG. When using monoclonal antibodies, cells were sequentially incubated with monoclonal antibody and Alexa-Fluor 488-conjugated anti-mouse IgG. All incubations were performed in PBS with 0.1% casein for 30 min at room temperature. For each sample, IgG binding was expressed as the geometric mean fluorescence intensity (MFI) for infected erythrocytes (ethidium bromide positive), after subtracting MFI for uninfected erythrocytes. Sera from non-exposed donors and positive control samples were included in each assay.

Rabbit antiserum R1945 was generated by immunization of rabbits with CS2-infected erythrocytes; it has been previously shown to recognize var2csa-PfEMP1 (Elliott *et al*., 2005). Human serum samples used in assays were collected from malaria-exposed pregnant women who were residents of the Madang Province, Papua New Guinea (PNG), presenting for routine antenatal care at the Modilon Hospital, Madang from 2001 to 2002 (Beeson *et al*., 2007). Serum from five Australian residents were included as controls. Informed consent was given by all donors and ethical clearance was obtained from the Medical Research Advisory Committee, Department of Health, PNG, and the Human Research Ethics Committee of the Walter and Eliza Hall Institute.

### Ferrimagnetic microbeads and binding

Ferrimagnetic beads (solid Fe_3_O_4_, 2.3 μm) were prepared as previously described (Puig-de-Morales-Marinkovic *et al*., 2007). Briefly, beads were coated with Concanavalin A (ConA, Sigma, St Louis, MO, USA) by incubating overnight at 4°C in ConA solution (1 mg ml^−1^). ConA is not blood group specific but has an affinity for terminal α-d-mannosyl and α-d-glucosyl residues. A wide variety of serum and transmembrane glycoproteins have a ‘core oligosaccharide’ structure, which includes α-linked mannose residues.

### Magnetic twisting cytometry with optical detection

The experimental set-up is described elsewhere (Fabry *et al*., 2001; 2003). Briefly, cells adherent to the bottom of a glass well with beads attached to their surface were placed on an inverted microscope (Leica DM IRBE, Leica Microsystems, Weitzler, Germany) and viewed under bright field with an oil immersion 63× objective and optically magnified 1.5×. Beads were first magnetized horizontally, then subjected to a oscillatory magnetic field in the vertical direction. This oscillatory field causes a specific torque on the bead. The lateral displacements of the bead in response to the oscillatory torque were tracked using a CCD camera (JAI CV-M10, Gloatrup, Denmark) with an exposure time of 0.1 ms and acquisition frequency of 24 Hz. The bead position was computed using an intensity-weighted centre-of-mass algorithm (Fabry *et al*., 2001).

### Mechanical tests

Clean, 35 mm glass-bottom wells (MatTek, Ashland, MA, USA) were covered with 0.1 mg ml^−1^ poly-l-lysine (Sigma, St Louis, MO, USA) for 5 min at room temperature. *P. falciparum* erythrocytes were allowed to adhere for 10 min to the wells before the beads were added. Measurements were performed at oscillatory frequencies between 10^−1^ and 10^2^ Hz. As described previously, heterodyning was used when the sinusoidal forcing frequency was greater than 4 Hz (Fabry *et al*., 2001). All measurements using infected cells were performed at trophozoite stage, as they were clearly recognized under the microscope.

### Measurement of the complex viscoelastic modulus and statistical analysis

Storage modulus or stiffness, g′, and loss modulus or friction, g″, were extracted from the torque and corresponding lateral bead displacement as described elsewhere (Fabry *et al*., 2003; Puig-de-Morales-Marinkovic *et al*., 2007).

We checked for significance between the three groups (CS2, CS2Pf332trunc and CS2ΔPf332) using two different approaches. The first approach was focusing upon the stiffness responses at one particular frequency (0.75 Hz). We applied a Student's *t*-test between CS2 and CS2Pf332trunc, and between CS2 and CS2ΔPf332. The second approach was extending the comparison between stiffness responses across frequencies. We fit the mean data for each group to the model G*(*s*) = *A* + *Bs*^*α*^, where G*(*s*) is the complex elastic modulus in Laplace space (see for details Puig-de-Morales-Marinkovic *et al*., 2007). This model reflects the behaviour seen in Fig. 8A. Within each group we also estimated the standard error of the parameter A, because it is the dominant term describing the data in Fig. 8Aand in particular describing the stiffness data. We compared both CS2Pf332trunc and CS2ΔPf332 with the parental line CS2 by examination of the *z*-statistics for differences in the parameter A. All data presented are geometric means and SD or SE.
