# Supplementary material for: Analysis of structure and function of the giant protein Pf332 in Plasmodium falciparum
Source: Mol Microbiol. 2008 Nov 6;71(1):48–65. doi: 10.1111/j.1365-2958.2008.06508.x (PMC2680261; doi:10.1111/j.1365-2958.2008.06508.x)
Supplement: Supplementary file 1 [file mmi0071-0048-SD1.pdf]

Supporting Information for Hodder and Maier et al.

Fig. S1. Sequence alignment between EBA-175 and Pf332.

```
EBA-175    DKNSVDTNTKVWECKKPYKLSTKDVCVPPRRQELCLGNIDRIYDKN--LL
Pf332      --INNKDSSTEWNCKE------DVGCVPPRRQNLNMERLDNENEDSVPDF

EBA-175    MIKEHILAIAIYESRILKRKYKNKDDKEVCKIINKTFADIRDIIGGTDYW
Pf332      MKKTFYLAAAGEGKKLREKHDE--SCDEFCDAWNRSLADYKDIFQGKDMW

EBA-175    NDLSNRKLVGKINTNSNYVHRNKQNDKLFRDEWWKVIKKDVWNVISWVFK
Pf332      ND----GKYGEAKNHIKNAFGDMNNRKTMLNEIEKGIKDETFSRENGLDV

EBA-175    DKTVCKEDDIENIP-QFFRWFSEWGDDYCQDKTKMIETLKVECKEKPCED
Pf332      CKSQCEERSRDDTEDQFLRFFAEWEEEFCDGLNKHEEQLKS-C----TKD

EBA-175    DNCKRKCNSYKEWISKKKEEYNKQAKQYQEYQKGNNYKMYSEFKSIKPEV
Pf332      INCDIKCSNFKDWLETKKDEYDIQSRVFEKKYANDNKSKHL--------N

EBA-175    YLKKYSEKCSNLNFEDEFKEELHSDYKNKCTMCPEV
Pf332      YLKEGMNKCKVKNPEMVFKSG--FANVAECRNLNVE
```

Fig. S2 Hodder and Maier et al.

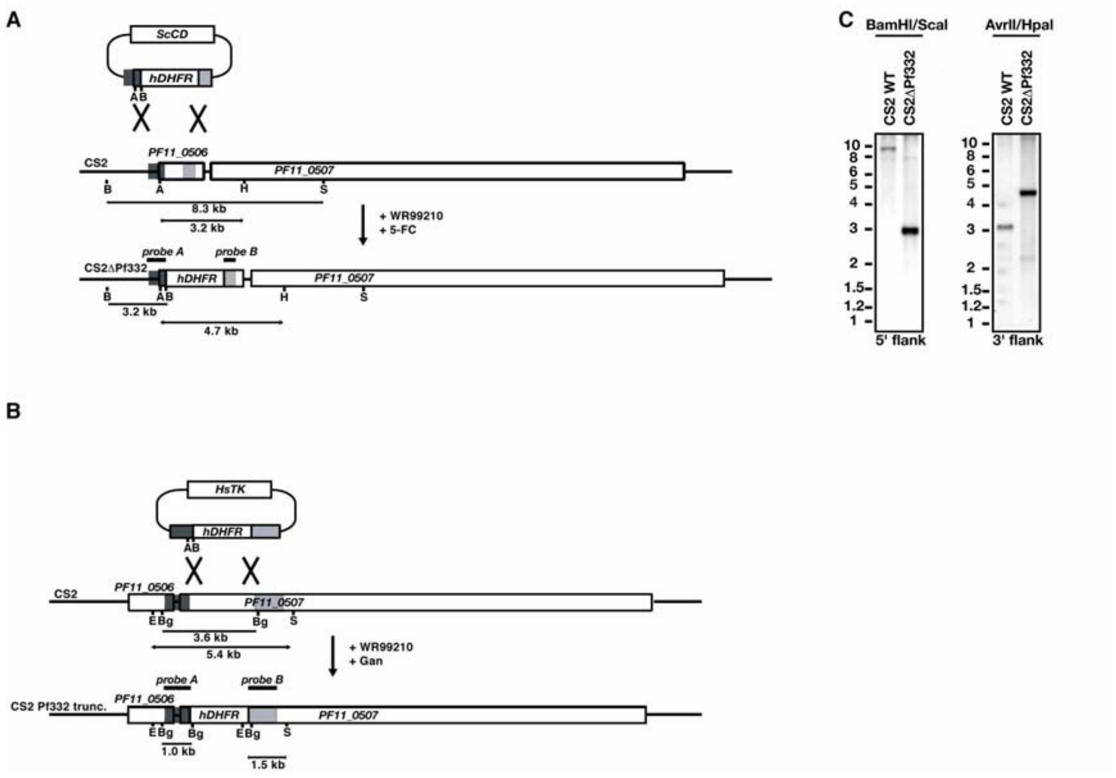

Fig. S2. **Scheme for disruption and truncation of the *Pf332* gene in *P. falciparum*.** (A) Schematic representation of the plasmid and structure of disrupted *Pf332* CS2DPf332 and CS2Pf332trunc. The *Pf332* gene was disrupted by insertion of the *hDHFR* gene cassette via homologous double crossover recombination to generate CS2DPf332. Restriction sites used for Southern blot analysis (A, *Avr* II: B, *Bam* HI: H, *Hpa* I: S, *Sca* I) and the predicted fragment sizes are shown in kilobases (kb). (B) Schematic representation of the plasmid and structure of truncated *Pf332* to generate CS2Pf332trunc. The *Pf332* gene was truncated by inserting the *hDHFR* cassette via homologous double crossover recombination, which resulted in the expression of the first (PF11_0506) and some of the second exon (PF11_0507) of the *Pf332* gene. Restriction sites used for Southern blot analysis were: Bg, *Bgl* II: E, *Eco*RI: S, *Sca* I. The predicted fragment sizes are shown in kilobases (kb). (C) Southern blot analysis of *Pf332* in CS2 and CS2DPf332. Genomic DNA was digested with *Bam* HI/*Sca* I or *Avr* II/*Hpa* I and probed with the 5’ (probe A) and 3’ (probe B) targeting sequences respectively. Predicted sizes for the hybridizing fragments using the 5’ probe were: CS2, 8.3 kb; CS2ΔPf332, 3.2 kb; and plasmid, 2.7 kb. Predicted sizes for the 3’ probe were: CS2, 3.2 kb; CS2ΔPf332, 4.7 kb; and plasmid, 2.8 kb.

Fig. S3 Hodder and Maier et al.

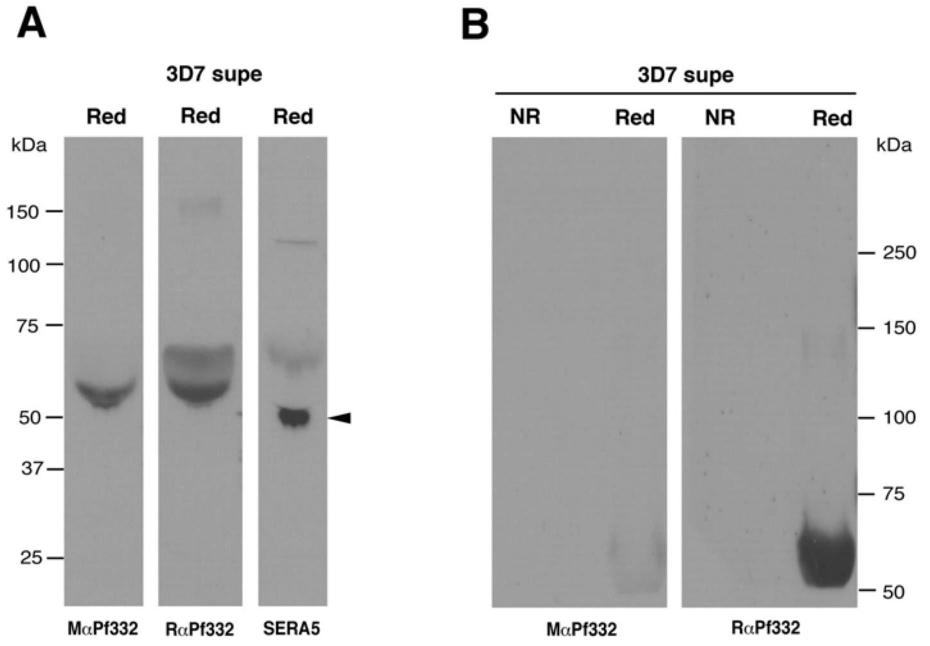

Fig. S3. **The Pf332 DBL protein is not detected in 3D7 culture supernatants.** *A*, A mouse monoclonal antibody and rabbit antiserum to Pf332 does not detect any Pf322 DBL protein or processed products in supernatants collected from parasites after schizont rupture and merozoite invasion. In contrast, rabbit antibodies to SERA5 detect the protein at ~50 kDa. The large diffuse area of reactivity around 68 kDa in the anti-Pf332 tracks, is a cross-reaction with albumin present in culture supernatant. *B,* The same anti-Pf332 antibodies used in panel A were used to probe both non-reduced (NR) and reduced (Red) proteins in 3D7 supernatant. Proteins were run to allow detection of large molecular weight proteins. Once again, the albumin cross-reaction is seen in the reduced track probed with rabbit anti-Pf332 antibodies.
